# Comparative Genomic Insights into Bacterial Induction of Larval Settlement and Metamorphosis in the Upside-Down Jellyfish *Cassiopea*

**DOI:** 10.1128/msphere.00315-22

**Published:** 2023-05-08

**Authors:** Aki Ohdera, Khushboo Attarwala, Victoria Wu, Rubain Henry, Henry Laird, Dietrich K. Hofmann, William K. Fitt, Mónica Medina

**Affiliations:** a Department of Biology, Pennsylvania State University, State College, Pennsylvania, USA; b National Museum of Natural History, Smithsonian Institute, Washington, DC, USA; c University of Wisconsin, Madison, Wisconsin, USA; d Ruhr-University, Bochum, Germany; e Odum School of Ecology, University of Georgia, Athens, Georgia, USA; f Smithsonian Tropical Research Institute, Smithsonian Institute, Washington, DC, USA; Nanjing University of Chinese Medicine

**Keywords:** *Cassiopea*, larval settlement, metamorphosis, *Pseudoalteromonas*

## Abstract

Bacteria are important mediators of the larval transition from pelagic to benthic environments for marine organisms. Bacteria can therefore dictate species distribution and success of an individual. Despite the importance of marine bacteria to animal ecology, the identity of inductive microbes for many invertebrates are unknown. Here, we report the first successful isolation of bacteria from natural substrates capable of inducing settlement and metamorphosis of the planula larvae stage of a true jellyfish, the upside-down jellyfish *Cassiopea xamachana*. Inductive bacteria belonged to multiple phyla, with various capacity to induce settlement and metamorphosis. The most inductive isolates belonged to the genus *Pseudoalteromonas*, a marine bacterium known to induce the pelago-benthic transition in other marine invertebrates. In sequencing the genome of the isolated *Pseudoalteromonas* and a semiinductive *Vibrio*, we found biosynthetic pathways previously implicated in larval settlement were absent in *Cassiopea* inducing taxa. We instead identified other candidate biosynthetic gene clusters involved in larval metamorphosis. These findings could provide hints to the ecological success of *C. xamachana* compared to sympatric congeneric species within mangrove environments and provide avenues to investigate the evolution of animal-microbe interactions.

**IMPORTANCE** The pelagic to benthic transition for the larvae of many marine invertebrate species are thought to be triggered by microbial cues. The microbial species and exact cue that initiates this transition remains unknown for many animals. Here, we identify two bacterial species, a *Pseudoalteromonas* and a *Vibrio*, isolated from natural substrate that induce settlement and metamorphosis of the upside-down jellyfish *Cassiopea xamachana*. Genomic sequencing revealed both isolates lacked genes known to induce the life history transition in other marine invertebrates. Instead, we identified other gene clusters that may be important for jellyfish settlement and metamorphosis. This study is the first step to identifying the bacterial cue for *C. xamachana*, an ecologically important species to coastal ecosystems and an emerging model system. Understanding the bacterial cues provides insight into marine invertebrate ecology and evolution of animal-microbe interactions.

## INTRODUCTION

Microbe-animal interactions have shaped the evolution of metazoans, likely facilitating the emergence of multicellularity and the diversification of animal lineages (1, 2). In particular, larval settlement, the behavioral migration from the planktos to the benthos of some marine invertebrates, and metamorphosis, a developmental transition of life stage, is often triggered by microbe-animal interactions (3–6). Microbes thus dictate developmental timing of marine invertebrates, with implications for ecological and population dynamics of the animal. While abiotic factors, including light, temperature, salinity, and substrate contribute to site selection for settlement, bacterial cues are found to be increasingly important to the life history of many marine animals (7–9).

Bacterial biofilms found on substrates are often sources of these cues, which can vary from water soluble compounds to physical cues (10–13). Detection of bacterial cues allow larvae to select appropriate habitats for subsequent adult survival, and can often be highly specific (14, 15), with larvae delaying settlement for prolonged periods when cues are undetected (16). Bacteria-associated site selection may also benefit the larvae, as some secondary metabolites exhibit anti-bacterial and anti-fungal properties, potentially acting as a “secondary immune system” for the animal (17, 18). Despite the ecological and evolutionary importance of microbes to marine benthic communities, few bacterial inducers of larval settlement and metamorphosis have been identified. Moreover, identification of the cues responsible for settlement and metamorphosis have been limited. Determining the diversity of cues and the sources of these cues is important to understanding the drivers that shape marine invertebrate life history.

In the upside-down jellyfish *Cassiopea xamachana*, microbial cues are predicted to initiate larval settlement and metamorphosis. Neumann (1979) showed *Cassiopea* metamorphose in response to *Vibrio* sp. under laboratory conditions. Cholera toxin isolated from V. cholerae was also found to induce settlement and metamorphosis (19), and an extract from collagen digested by V. alginolyticus was also found to be inductive (20). In their natural environment, brooded *C. xamachana* larvae are released into the water column and will often settle preferentially onto the underside of degrading mangrove leaves (7, 21). When treated with antibiotics, larval settlement and metamorphosis is abolished in response to mangrove leaves. These findings suggest bacteria associated with degrading leaves may be a source of the inductive cue of *Cassiopea*. In a follow-up study, Fleck and Fitt (21) showed a water soluble, 5.8 kDa proline-rich peptide extracted from degraded mangrove leaves to be an inducer of settlement in *C. xamachana*. However, the originating bacterial source of the cue, and whether the cue is a by-product of leaf degradation, remains unknown.

A large body of work has offered clues (14, 15) for how settlement and metamorphosis occurs through animal-microbe interactions. Yet, we do not fully understand the extent of the specificity underlying the developmental transition of pelagic larvae to benthic juveniles. Here, we investigate substrate preference of the upside-down jellyfish *Cassiopea xamachana* found in mangrove environments. We report the first isolation of inductive bacteria of the upside-down jellyfish from natural substrate. We isolated bacterial species from mangrove leaves and sand to identify bacteria capable of inducing the pelago-benthic transition in *C. xamachana.* Comparative genomics of bacterial isolates capable of inducing settlement and metamorphosis revealed potential mechanisms underlying induction in *C. xamachana*. Determining larval settlement in *Cassiopea* can further our understanding of animal-microbe interaction dictating development. In addition, the development of the jellyfish as a model to study host-microbe interactions necessitates identification of bacterial inducers of larval settlement.

## RESULTS

### Bacteria isolated from field collected substrate induce larval settlement of *Cassiopea*.

Fleck and Fitt previously suggested the natural cue for settlement and metamorphosis originates from decomposing plant matter (7). In order to determine whether plant degradation by associated bacteria is necessary, we performed a settlement assay with both biotic and abiotic substrates. Substrates were collected at both shallow (1 to 2 m) and deep (3 to 4 m). Sampling depth of substrate was tested as *C. xamachana* medusae are often found in shallower depths.

**FIG 1:**
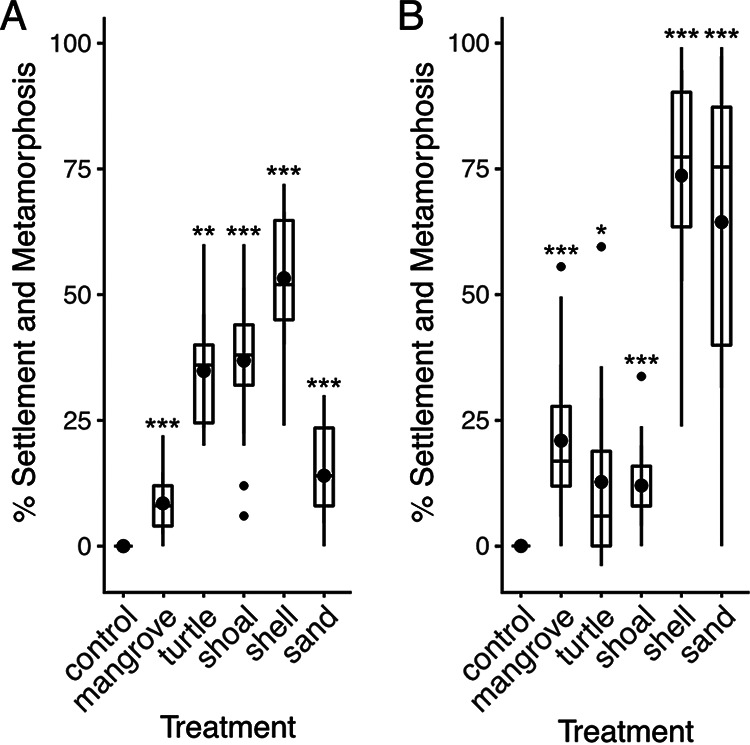
Rates of settlement and metamorphosis of *Cassiopea xamachana* larvae after 72 h in response to natural substrate. Natural substrates were collected from deep (A) and shallow sites (B). Settlement rates of treated larvae were compared to control larvae in filtered seawater. *, *P*-value < 0.05; **, *P*-value < 0.005; ***, *P*-value < 0.0005 (Kruskal-Wallis, *post hoc* Wilcoxon test).

We found larvae significantly settled and metamorphosed under laboratory conditions in response to both biotic and abiotic substrates collected at both shallow and deeper depths (Fig. 1A and B, Fig. S1) (*P*-value < 0.05, Kruskal-Wallis, *post hoc* Wilcoxon test). Larvae of *C. xamachana* responded to mangrove leaves, a substrate previously shown to be highly inductive (7). In our experiment however, *C. xamachana* larvae exposed to mangrove leaves exhibited relatively low settlement rates compared to other substrates. Mollusk shells collected from both shallow and deep depths led to the highest settlement and metamorphosis rate. Sand collected from shallow depth was also highly inductive. When we compared substrate type between two depths, the effect of depth appeared to be substrate dependent. Abiotic substrate collected from shallow depth was more inductive compared to those collected from the deep site (Fig. S1B). In general, biotic substrates were more inductive when collected from deep sites. The difference in induction by mangrove leaves was not significantly different between depths.

10.1128/msphere.00315-22.1 FIG S1 *Cassiopea xamachana* larvae settlement and metamorphose in response to biotic and abiotic factors. (A) Natural substrates used in the study were collected from deep (3–4 meters) and shallow (1–2 meters) site B in the Upper Florida Keys. Mangrove leaves and sand sampled to isolate associated bacteria were collected from shallow site B. Samples of degrading mangrove leaves used for the 16S amplicon sequencing analysis were collected from all four sites. (B) Larvae differentially responded to substrate collected from deep and shallow sites. Substrate specific pair-wise testing of significance in settlement rate between deep and shallow sites was performed with the Kruskal-Wallis analysis with *post hoc* Wilcoxon test. *, *P*-value < 0.05. n.s., not significant. Download FIG S1, TIF file, 0.6 MB. Copyright © 2023 Ohdera et al. 2023 Ohdera et al. https://creativecommons.org/licenses/by/4.0/ This content is distributed under the terms of the Creative Commons Attribution 4.0 International license.

These data suggest degradation of plant material is not necessary for *C. xamachana* as a source for the settlement cue. If the cue is bacterial in origin, inductive microbes are likely found associated with multiple substrate types. We therefore sought to determine whether *C. xamachana* settle and metamorphose in response to multiple bacterial species.

In order to determine whether microbes associated with inductive substrates can induce settlement and metamorphosis in *C. xamachana*, we isolated bacteria from newly collected mangrove leaves and sand collected from shallow sites. Despite the response of larvae to mangrove leaves, it was selected as a substrate based on previous reports of their inductive capacity, while sand was chosen based the results obtained for this study. We were successful in isolating a broad diversity of bacterial taxa from both sand and degrading mangrove leaves.

**FIG 2:**
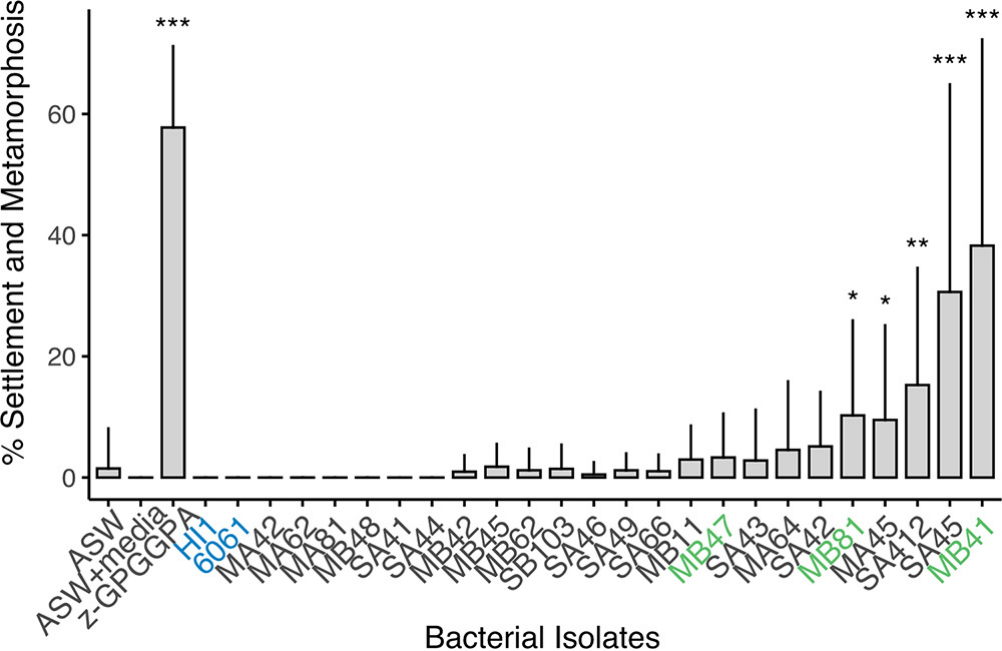
Rates of settlement and metamorphosis of *Cassiopea xamachana* larvae in response to monoculture of bacterial isolates. Larvae exposed to monoculture of bacterial isolates were compared to larvae kept in filtered artificial seawater (ASW), ASW in wells preincubated with marine broth, and an inductive hexapeptide (Z-GPGGPA). Settlement and metamorphosis was scored after 48 h. *, *P* value < 0.05; **, *P*-value < 0.005; ***, *P*-value < 0.0005 (Kruskal-Wallis, *post hoc* Wilcoxon test). Strains of Pseudoalteromonas luteoviolacea (HI1, 6061) previously found to induce settlement and metamorphosis of other marine invertebrate species were also tested (shown in blue). *Pseudoalteromonas* species isolated for this study are labeled in green. Error bars indicated standard deviation between trials.

Settlement rate of *C. xamachana* larvae exposed to the bacterial isolates varied (Fig. 2). Rates of larval settlement and metamorphosis ranged from 0% to nearly 40% over 2 days. In comparison, the small peptide inducer Z-GPGGPA used here as a positive-control induced settlement and metamorphosis at an average rate of 57.8%. Larvae did not significantly settle and metamorphose in the absence of an inducer. Filtered artificial seawater (FSW) and FSW in assay plates preconditioned with bacterial growth media resulted in an average induction of 1.49% and 0%, respectively. In particular, isolate MB41 and SA45 exhibited the greatest metamorphic induction among the tested isolates, with an induction rate of 38.25% and 32.07%, respectively. All other isolates either failed to induce settlement or did not exceed 10% settlement on average. However, five of the isolates elicited a significant settlement and metamorphosis response in *C. xamachana* larvae compared to FSW (Kruskal-Wallis, *post hoc* Wilcoxon test, *P*-value < 0.05) (Fig. 2).

**FIG 3:**
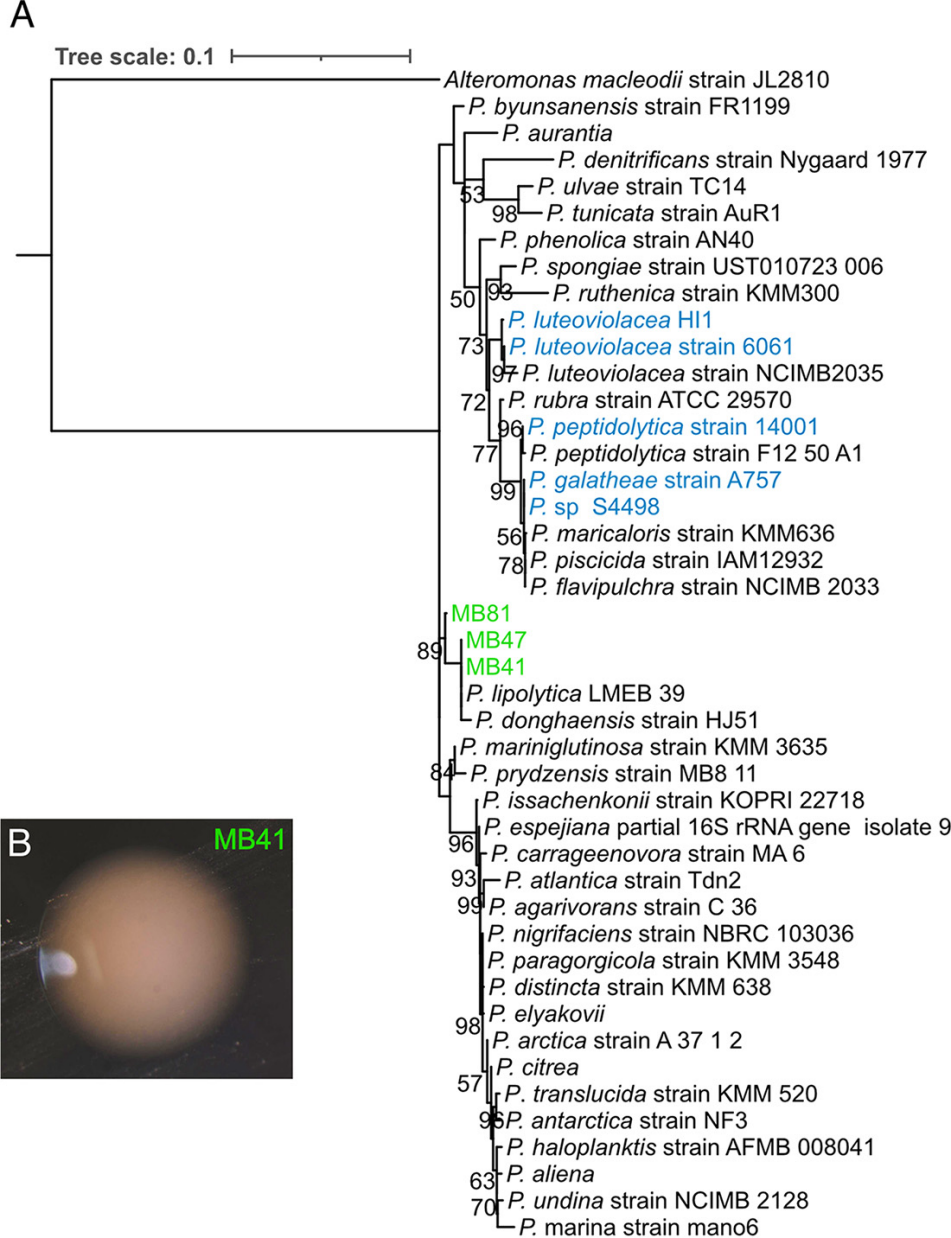
Phylogenetic reconstruction of the genus *Pseudoalteromonas* using the 16S rRNA gene. (A) The maximum likelihood tree was constructed with IQ-TREE with ultrafast bootstrap approximation (*n* = 2000). Branch supports are indicted on the tree. Isolates of *Pseudoalteromonas* collected during this study are indicated in green. *Pseudoalteromonas* species previously found to induce settlement and metamorphosis of other marine invertebrates are shown in blue. (B) A colony of isolate MB41 (*Pseudoalteromonas* sp.) growing on marine agar.

Taxonomic classification using the 16S rRNA gene revealed the inductive isolates belonged to diverse bacterial groups (Text S1). The five most highly inductive taxa were identified as belonging to Pseudoalteromonadaceae, Sphingomonadaceae, and Rhodobacteraceae. Isolates MB41, MB47, and MB81 belonged to genus *Pseudoalteromonas*, closely related to Pseudoalteromonas lipolytica and *P. donghaensis* (Fig. 3). A BLASTn analysis between the *P. lipolytica* (NCBI Accession NR_116629.1) and MB41 16S rRNA gene resulted in a sequence similarity of 99.9%. Given that Pseudoalteromonas luteoviolacea is a well characterized settlement inducer of the polychaete worm *Hydroides elegans* (10, 22, 23), we also tested whether strains of *P. luteoviolacea* induced settlement in *C. xamachana* larvae. Monoculture of *P. luteoviolacea* HI1 and 6061 failed to induce settlement and metamorphosis. In prior studies a *Vibrio* was found to induce settlement in *C. andromeda* (24, 25). Isolate MA64, with a 16S rRNA sequence similarity of 99.3% to Vibrio alginolyticus (NCBI Accession NR_044825.2), also induced settlement and metamorphosis but was not significant compared to filtered artificial seawater (*P* > 0.05).

### Inductive isolates are rare in degrading mangrove leaves.

**FIG 4:**
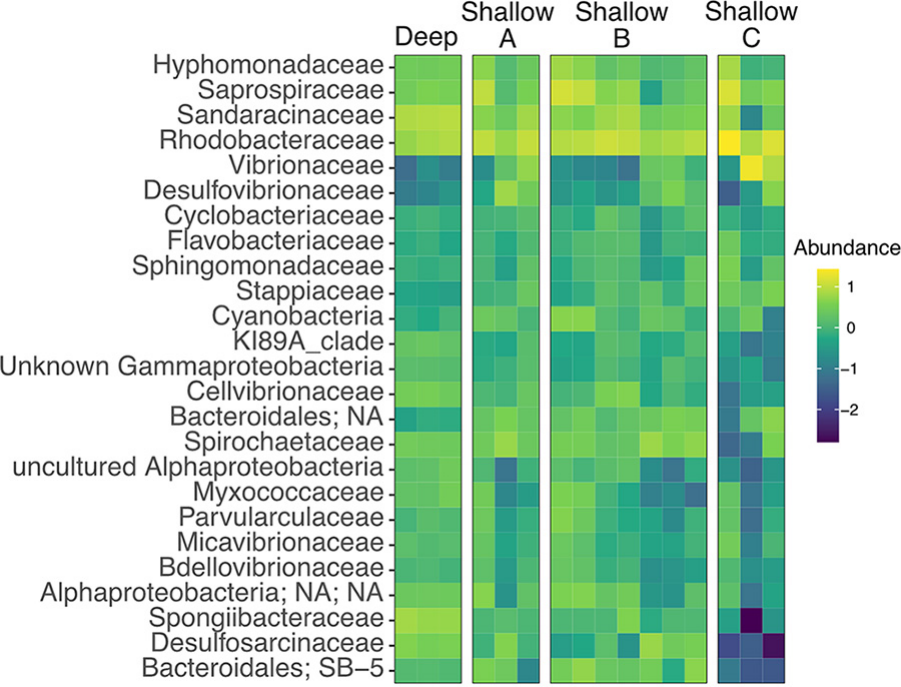
Heatmap of the top 25 most abundant family of bacteria identified from degrading mangrove leaves. Community composition of mangrove leaves were determined by shotgun sequence the V4 region of the 16S rRNA gene. Taxonomic identification of the assembled sequences was performed using Qiime2 and the Silva database (release 138). Abundance was determined with Qiime2 and Log_10_ transformed for visualization. Degrading mangrove leaves were collected from one deep site (3–4 m) and three shallow (1–2 m) sites.

To determine whether the assayed isolates capable of inducing settlement and metamorphosis were commonly associated with the inductive substrate, we performed amplicon sequencing of degrading mangrove leaves from three different sites. We found the microbiome of mangroves leaves to be highly diverse, and identified approximately 3,000 amplicon sequence variants (ASVs) with greater than 5 reads across the samples. The most abundant groups comprised roughly 25% of the total reads per sample. The five most common family of bacteria associated with mangroves leaves were Vibrionaceae, Sandaracinaceae, Rhodobacteraceae, Saprospirae, and Spirochaetaceae. Community composition differed significantly between sites (Permanova, 9999 permutations, *P*-value < 0.05), including between the deep and shallow sites (Fig. 4, Table S1). However, community composition was not significant when Benjamini-Hochberg FDR correction was applied for pairwise comparisons between sites.

10.1128/msphere.00315-22.3 TABLE S1 Permanova analysis of community composition between shallow (A-C) and deep sites. Permanova analysis was performed with *beta-group-significance* function of Qiime2*^a^* *^a^*Q-values were generated by applying Benjamini-Hochberg FDR correction to the *P*-value statistic. Download Table S1, TIF file, 0.3 MB. Copyright © 2023 Ohdera et al. 2023 Ohdera et al. https://creativecommons.org/licenses/by/4.0/ This content is distributed under the terms of the Creative Commons Attribution 4.0 International license.

Using a predefined cutoff of 98% sequence similarity, we identified ASVs matching the V4 region of the 16S rRNA gene of the bacterial isolates. We found that inductive isolates were rare within the mangrove microbiome for all sites (Text S2). In general, inductive taxa composed less than 1% of the total reads generated for each sample. Of the inductive isolates, including those that elicit low settlement rates in *C. xamachana* larvae, a *Vibrio* sp. was the only ASV present that comprised greater than 1% of total reads in the mangrove leaf microbiome. Based on the bioassay results and the 16S amplicon sequencing results, we sequenced select taxa to study the genetic basis for the induction of larval settlement and metamorphosis.

### Genome analysis of inductive isolates.

The genus *Pseudoalteromonas* has been implicated in larval settlement and metamorphosis across many marine species (3, 26, 27). We therefore sequenced the genome of the highly inductive *Pseudoalteromonas* isolate MB41 to identify potential genomic signatures associated with induction of larval settlement. We generated a 4.85 Mb assembly with 4452 predicted coding genes (Table S2). We determined the genome completeness to be 100%, with 1.03% contamination. Average nucleotide identity (ANI) was greater than 98% compared with *P. lipolytica* strain CSB02KR and *P. donghaensis* strain HJ51 (Text S3), supporting the 16S rRNA gene analysis. Using the genome, we first searched the *Pseudoalteromonas* genome assembly for genes known to induce settlement and metamorphosis.

10.1128/msphere.00315-22.4 TABLE S2 Assembly statistics for *Pseudoalteromonas* MB41 and Vibrio MA64. *De novo* genome assembly was performed with the A5-miseq pipeline*^a^* *^a^*Assembly statistics were computed with RAST. Genome completeness and contamination was calculated with CheckM. Download Table S2, TIF file, 0.3 MB. Copyright © 2023 Ohdera et al. 2023 Ohdera et al. https://creativecommons.org/licenses/by/4.0/ This content is distributed under the terms of the Creative Commons Attribution 4.0 International license.

**FIG 5:**
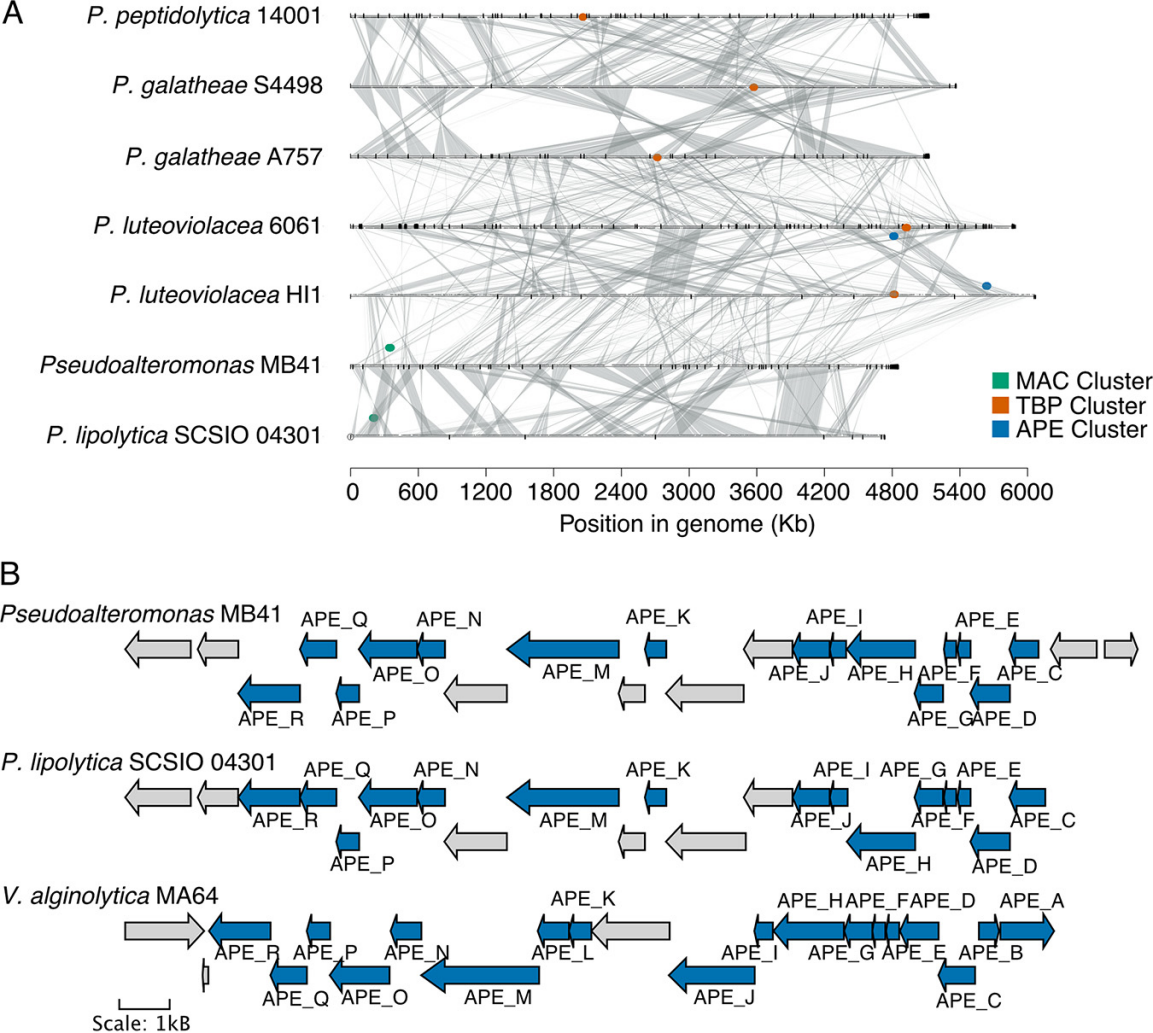
Comparative genomics of *Pseudoalteromonas* spp. (A) Genome synteny map of *Pseudoalteromonas* spp. colored points indicate genomic position of biosynthetic gene clusters. MAC = Tailocins. TBP = Tetrabromopyrrole. APE = Aryl polyene biosynthesis clusters. (B) Biosynthetic gene cluster of aryl polyene found in *Pseudoalteromonas* MB41, Pseudoalteromonas lipolytica, and *Vibrio* MA64. Genes previously confirmed to be involved in biosynthesis of aryl polyene are shown in blue.

Pseudoalteromonas luteoviolacea produces tailocins, a well characterized inductive cue of settlement and metamorphosis of the marine polychaete tubeworm *Hydroides elegans* (10, 11, 28). Composed of viral sheath components, tailocins are encoded by the *MAC* protein gene cluster (4). In our isolate MB41 as well as in *P. lipolytica*, the tailocin gene cluster was absent revealing that MB41 induces settlement via other modes (Fig. 5A, Fig. S2).

10.1128/msphere.00315-22.2 FIG S2 Tetrabromopyrrole synthesizing *bmp* gene cluster identified from pigmented *Pseudoalteromonas* species. Download FIG S2, TIF file, 0.7 MB. Copyright © 2023 Ohdera et al. 2023 Ohdera et al. https://creativecommons.org/licenses/by/4.0/ This content is distributed under the terms of the Creative Commons Attribution 4.0 International license.

In addition to tailocins, the *bmp*1-10 biosynthetic gene cluster (BGC) of *Pseudoalteromonas* encodes a set of enzymes known to produce the brominated compound tetrabromopyrrole. Tetrabromopyrrole was first identified to induce settlement and metamorphosis of corals (13, 29). Species in our analysis known to produce tetrabromopyrrole possessed a minimum of 6 of the 10 genes within the BGC, while all *bmp* genes were absent from the *P. lipolytica* and MB41 genomes (Fig. 5A). We therefore predicted additional BGCs with antiSMASH (30). Unlike the genomes of the pigmented *Pseudoalteromonas*, which includes *P. luteoviolacea*, few BGCs were predicted from our isolate MB41. Only two putative BGCs, an aryl polyene (APE) and ribosomally synthesized and posttranslationally modified peptide (RiPP) biosynthesis clusters were identified from MB41. APEs are polyunsaturated carboxylic acids, often found in the outer membranes of host-associated bacteria. BLAST alignments of the MB41 APE genes against previously described clusters suggested that the MB41 cluster is most similar to the flexirubin-like APE cluster found in Vibrio fischeri (31), although the biosynthetic product of the MB41 cluster remains unknown (Table S3). Of the *Pseudoalteromonas* genomes surveyed, APE clusters were only found in MB41 and *P. lipolytica* (Fig. 4). The RiPP cluster in MB41 was characterized by a DUF692, previously linked to the production of bacteriocins, or antibacterial peptides (32). Although the exact product from the RiPP clusters are unknown, we found these BGCs are also present in both the *P. luteoviolacea* HI1 and *P. lipolytica* genomes (Table S4).

10.1128/msphere.00315-22.5 TABLE S3 Genes that comprise the aryl polyene (APE) cluster identified in *Pseudoalteromonas* sp. MB41, Pseudoalteromonas lipolytica, and *Vibrio* sp. MA64*^a^* *^a^*Presence of APE genes were determined by antiSMASH prediction and confirmed by BLAST against the APE cluster identified in E. coli, where the IDs are borrowed from (Johnston et al. 2021). Download Table S3, TIF file, 1.1 MB. Copyright © 2023 Ohdera et al. 2023 Ohdera et al. https://creativecommons.org/licenses/by/4.0/ This content is distributed under the terms of the Creative Commons Attribution 4.0 International license.

10.1128/msphere.00315-22.6 TABLE S4 Results of the secondary metabolite and biosynthetic gene cluster analysis performed with antiSMASH (ver. 6.0). Download Table S4, TIF file, 0.7 MB. Copyright © 2023 Ohdera et al. 2023 Ohdera et al. https://creativecommons.org/licenses/by/4.0/ This content is distributed under the terms of the Creative Commons Attribution 4.0 International license.

As the most abundant inductive genus, albeit not the highest inducer, we also sequenced the genome of the *Vibrio* isolate MA64 to produce a 5.5 Mb assembly with 5256 coding sequences (Table S2). MA64 was highly similar to V. alginolyticus strain HYV1, with an ANI value greater than 98% (Text S4). Similar to *Pseudoalteromonas* isolate MB41, the MA64 genome lacked both tailocin and tetrabromopyrrole BGCs. Secondary metabolite cluster prediction resulted in six putative clusters, including a putative APE (Fig. 5B) and RiPP cluster (Table S4). In addition, we identified a cluster most similar to vibrioferrin, ectoine, and betalactone, as well as a 100% match to the vanchrobactin BGC. Like *Pseudoalteromonas* isolate MB41, the MA64 APE cluster was most similar to the flexirubin-like cluster found in V. fischeri (Fig. 5B). The RiPP cluster of MA64 was also characterized by a DUF2063 and likely the core peptide.

## DISCUSSION

Bacteria have been shown to be a source of larval settlement cues for marine invertebrate species. However, the identity and specificity of bacteria capable of inducing settlement and metamorphosis of invertebrates remain largely unknown (25, 29, 33–37). We found larvae of *C. xamachana* to settle and metamorphose when exposed to taxa belonging to multiple phyla (Fig. 1). Two of the inductive isolates were identified as belonging to the marine genus *Pseudoalteromonas* (MB41) and the nonpathogenic *Vibrio* closely related to V. alginolyticus (MA64). The latter has previously been shown to induce settlement and metamorphosis in *C. andromeda*, but this is the first recording in which a jellyfish settlement inducing species of *Vibrio* was isolated from a natural substrate. Additionally, induction of settlement and metamorphosis of a true jellyfish by a *Pseudoalteromonas* has yet to be described, despite the ubiquity of the species in the literature as an inducer of larval metamorphosis (29).

The discovery of a *Pseudoalteromonas* as an inducer (MB41), while another is non-inductive (*P. luteoviolacea)*, highlights the importance of specificity and mode of induction underlying the association. Our finding and those of others that inductive bacteria are rare in substrate associated microbial communities further emphasizes the importance of mechanisms that facilitate the specificity (Fig. 3) (38, 39). In fact, some animal species preferentially settle in proximity to rare microbial taxa (40). Larvae may also respond to multiple taxa or multiple cues, including bacterial species that may be difficult to culture but common within biofilm. This confounds the exact animal-microbe interaction that drives the pelago-benthic transition for *Cassiopea*. However, we were able to show that larvae respond to natural substrate under laboratory conditions where other factors such as light are controlled. Therefore, the difference in settlement rate between substrate collected from deep and shallow sites likely point to bacterial community composition as an important factor in dictating the larval transition. Furthermore, the response of *C. xamachana* larvae to different microbial taxa suggests redundancy in cues, which can be predicted to have ecological and evolutionary implications.

Our data also suggest a plant substrate is unnecessary for settlement of *C. xamachana* larvae and inductive bacteria do not require exogenous molecules as precursors to produce the settlement inducing molecule(s). Therefore, biosynthetic gene clusters (BGCs) found in inductive taxa could provide clues to the inductive molecule. In searching for inductive biosynthetic gene clusters, we found the *Pseudoalteromonas* isolate MB41 and *Vibrio* isolate MA64 genomes both lack genes responsible for tetrabromopyrrole biosynthesis, as well as tailocins in the case of *Pseudoalteromonas*, although the presence of the tailocin genes cluster is not predictive of settlement induction (41) (Fig. 5). Similar to previous findings, tailocin gene clusters were only found in *P. luteoviolacea*. The *bmp* gene cluster responsible for tetrabromopyrrole production typically contains 10 genes and is commonly found in pigmented *Pseudoalteromonas* species. The *bmp* gene cluster was absent from the genome of the unpigmented *Pseudoalteromonas* isolate MB41.

We also identified putative clusters responsible for aryl polyenes (APE) biosynthesis. APEs are polyketide derivatives, a class of pigments related to carotenoids and common in Gram-negative bacteria (42). Neither *Pseudoalteromonas* isolate MB41 and *Vibrio* isolate MA64 are considered pigmented, yet possess the BGCs (Fig. 5). MB41 belongs to the non-pigmented group within the *Pseudoalteromonas* genus (Fig. 2). Pigmented *Pseudoalteromonas* have been shown to prevent the attachment of organisms to substrates, including larvae and bacteria. Loss of pigmentation is associated with loss of the inhibitory function (43, 44). Of the four classes of aryl polyenes, the cluster found in MB41 and MA64 were most similar to flexirubin-like BGC found in Vibrio fischeri (Cimermancic 2014). As with other APEs, the flexirubin-like APE is thought to be antioxidative, but further investigation will be required to determine its involvement as an inductive metabolite of invertebrate settlement and metamorphosis.

Despite the importance of bacterial-larval interactions to animal evolution and ecology, we know little of the inductive cues responsible for larval settlement and metamorphosis. Given the wealth of information available through genomic data, understanding genomic features underlying settlement and metamorphosis can accelerate discovery of additional mechanisms driving the developmental transition. Understanding larval settlement provides information on species distribution and can aid in restoration efforts, particularly in the case of ecologically important scleractinian coral species (45).

## MATERIALS AND METHODS

### Larval collection.

Brooding females of *C. xamachana* were collected from Key Largo, FL (25.102204, -80.438708, 24.749522, -80.978341) (Fig. S1A). Eggs were removed from the females by gently pipetting seawater at the central brood vesicles and released embryos were collected into 0.22 μM filtered artificial seawater. Developing embryos were transferred to glass finger bowls containing antibiotic seawater to prevent fouling of the water leading to larval mortality (100 μg/mL neomycin, 130 μg/mL streptomycin). Embryos were left undisturbed for 48 h to allow development into mobile planulae. The planulae were transferred to fresh antibiotic seawater (ABS) and used for settlement assays within 24 h.

### Bacterial isolation.

Degraded mangrove leaves were collected from below mangrove stands and rinsed gently three times with 0.2 μm filtered seawater (FSW). 1 cm^2^ pieces were cut from the leaves and homogenized in 25 mL of FSW using an ethanol and flame sterilized mortar and pestle. Serial dilutions of the homogenates were made by transferring 0.5 mL of the diluted homogenate into 9.5 mL of FSW for 5 dilutions. 100 μL of each dilution was plated onto marine agar (Difco Marine Agar 2216) and incubated at 28°C for 24 h. Bacterial colonies were picked and preserved in FSW with 15% glycerol at −80°C.

### Identification of bacterial isolates.

The full length of the 16S rRNA gene of the bacterial isolates was sequenced using universal primer pair 27F and 1492R (46). Initially, bacterial stocks were plated onto marine agar and grown for 48 h. Colonies were picked and added directly to the PCR mix and the 16S gene was amplified using the following cycling conditions: 95 for 5 min, 94 for 1, 55 for 1:30, 72 for 2:00, 72 for 5:00, for 35 cycles. Sanger sequencing of amplicon products was performed at the Pennsylvania State University Sequencing Core Facility. Chromatograms were manually curated using MEGA, and sequences were compared to the NCBI nt database using BLASTn for taxonomic identification. Phylogenetic analysis of isolates belonging to the genus *Pseudoalteromonas* was performed as follows: First the 16S rRNA gene sequences were aligned with MAFFT (ver. 7.271) (47). Alignments were trimmed with BMGE (ver. 1.12) with default settings (48). IQ-TREE (ver. 1.6.12) was used for phylogenetic inference, with model selection performed using ModelFinder (49). Tree visualization and curation was performed with iTOL (ver. 6).

### Settlement assays.

For bioassays testing the inductive capacity of natural substrates, larvae were placed in 12-well plates with substrate collected from deep (25.128433, -80.443574) and shallow (25.102204, -80.438708) sites (Fig. S1). Assays were performed by providing 3 × 3 mm squares of plant substrates (mangrove leaf, turtle grass, shoal grass), several grains of sand, or small shell pieces to the larvae. Mangrove leaves and sea grass were visually inspected to confirm degradation as described by Fleck and Fitt (1999). Substrates were gently washed with filtered with seawater prior to their addition to the experimental plates. Control wells were filled with filtered artificial seawater (FSW). 6 wells with 6 larvae were prepared for each substrate type. Plates were kept undisturbed at 25°C in the dark. The larvae in plates were scored for settlement and metamorphosis at 72 h after exposure to the substrates.

Bioassays testing the inductive capacity of bacterial monoculture were prepared in 24-well plates by filling individual wells with 1 mL of sterile marine media (Difco Marine Broth 2216). Control wells were filled with filtered artificial seawater (FSW). A second negative control was included in which wells pretreated with marine media were left uninoculated and rinsed with FSW as described below. Wells containing media were inoculated with isolates by transferring a single colony to a well. Wells were inoculated in triplicate per isolate and were randomly assigned across multiple plates. Plates were incubated at 28°C for 24 h without shaking to allow the bacteria to grow and settle on the surfaces of the well. Wells were gently rinsed with 1 mL of FSW without disrupting the settled bacteria for a total of 2 washes at the end of the 24 h. Wells that were still turbid after the two washes were rinsed for a third time, or until the seawater remained clear. Wells were then filled with a total of 2 mL of 0.2 μm FSW. A positive control was included in the assay where the synthetic settlement and metamorphosis inducing peptide Z-GPGGPA (Bachem) were added at a final concentration of 1.5 × 10^−5^ M. Approximately 10 *C. xamachana* larvae kept in ABS for 48 h were placed in each of the wells. Plates were kept undisturbed at 25°C in the dark. The larvae were scored for settlement and metamorphosis at 24 and 48 h using a Stemi 305 stereo microscope (Zeiss).

Statistical analysis for both experiments was performed using R. Normal distribution of the data were tested using shapiro.test() function. Significance of variance was tested using the kruskal.test() function. A *post hoc* Wilcoxon signed rank test was performed using the pairwise.wilcox.test() function to determine statistical significance between treatment pairs (*P*-value cutoff = 0.05). For each treatment, rates of settlement and metamorphosis were compared to the filtered seawater control.

### 16S microbiome extraction and sequencing.

Degraded mangrove leaves were collected during the summers of 2013 and 2015 in Key Largo Florida from five different locations (Fig. S1A). Leaves were immediately stored at −20°C until further processing. DNA was extracted using the PowerSoil DNA isolation kit (Mobio) following the manufacturer protocol. The V4 region of the 16S rRNA gene was PCR amplified using the 515F (GTGCCAGCMGCCGCGCGGTAA)-806R (GGACTACHVGGGTWTCTAAT) primer pair (50). Sequencing was performed at the Joint Genome Institute (JGI) on an Illumina MiSeq with 300 bp paired-end run mode. Reads were analyzed using Qiime2 (ver. 2021.4). Reads were initially trimmed with Cutadapt (51), discarding any untrimmed reads. Reads were then further trimmed, followed by filtering and denoising performed with the Dada2 plugin of Qiime2 (Callahan et al. 2016) with the following parameters: –ptrun-len-f 250 –p-trunc-len-r 180 –p-trim-left-f 0 –p-trim-left-r 0 –p-trunc-q 2. Taxonomic assignment was performed with “feature-classifier classify-sklearn” using the Naïve Bayes classifier trained on the Silva_138_99%_505F/806R region specific database. Visualizations were performed with Qiime2R (ver. 0.99.6). The resulting feature table was filtered with the filter-features command with the following settings: –p-min-frequency 5 –p-min-samples 2. In order to determine the abundance of bacterial isolates collected from degrading mangrove leaves and sand, amplicon sequence variants (ASVs) were matched to the full-length 16S rRNA gene sequences using BLASTn (ver 2.5.0+). ASVs were determined to be a match with sequence identity greater than 98% (Supplemental File 1).

### Genome sequencing and analysis.

Bacterial isolates MB41 and MA64 were chosen for whole-genome sequencing based on their inductive capacities. Colonies were grown in marine media for 24 h. Genomic DNA was extracted following the JGI Bacterial DNA Isolation CTAB-2012 protocol (https://jgi.doe.gov/user-program-info/pmo-overview/). Illumina sequencing libraries were constructed using the Illumina TruSeq Nano DNA prep kit with a 550 bp insert size. Libraries were sequenced on the Illumina Miseq, generating 2.8 and 2.7 Gb of 300 bp paired-end sequence data for MB41 and MA64, respectively.

Draft genomes were *de novo* assembled using the A5-miseq pipeline using default settings (52). The A5-miseq pipeline performs reads trimming, error correction, contig assembly, and final scaffolding. Genes were annotated with Glimmer3.0 implemented through RAST (53). Completeness and contamination of the genomes were calculated using checkM (54), implemented in the Galaxy-based Protologger website (www.protologger.de) (55). Average nucleotide identity analysis was performed with fastANI (56) against genomes acquired from the NCBI Refseq database. The presence of tailocin encoding *mac* protein genes, tetrabromopyrrole gene cluster, and aryl polyene clusters were determined by performing BLASTp (ver 2.5.0+) against known gene clusters (57). Genomes and coding sequences of *Pseudoalteromonas* strains (GCF_000814765.1, GCF_001625655.1, GCF_012641745.1, GCF_004103285.1, GCF_005886105.1, GCF_000576675.1) used to compare presence of biosynthetic gene clusters were downloaded from the NCBI Refseq database. Secondary metabolite cluster prediction was performed with antiSMASH (ver. 6.0) (Blin et al. 2019). Visualizations were performed with Gene Graphics and Synima.

### Data availability.

Raw genomic sequencing reads, genomic assemblies of *Pseudoalteromonas* MB41 and *Vibrio* MA64, and raw 16S amplicon sequencing reads have been deposited in the NCBI/SRA database under the project accession PRJNA774281. Scripts and input data used for the bioassay and sequencing analysis are available via a GitHub repository (https://github.com/aohdera/Ohdera_et_al_2023).

10.1128/msphere.00315-22.7 TEXT S1 Bacterial isolates collected from highly degraded mangrove leaves and sand collected from a shallow (1–2 m depth) site. Inductive and non-inductive isolates and corresponding average induction rates are shown. Taxonomic placement of the isolates was performed through BLASTn against the NCBI nt database. Amplicon sequence variance with >98% sequence similarity are also provided. Download Text S1, XLSX file, 0.02 MB. Copyright © 2023 Ohdera et al. 2023 Ohdera et al. https://creativecommons.org/licenses/by/4.0/ This content is distributed under the terms of the Creative Commons Attribution 4.0 International license.

10.1128/msphere.00315-22.8 TEXT S2 Filtered feature table output from Qiime2 with relative abundance of individual amplicon sequence variants shown as a percentage. Download Text S2, XLSX file, 1.0 MB. Copyright © 2023 Ohdera et al. 2023 Ohdera et al. https://creativecommons.org/licenses/by/4.0/ This content is distributed under the terms of the Creative Commons Attribution 4.0 International license.

10.1128/msphere.00315-22.9 TEXT S3 Results of the average nucleotide identity analysis for *Pseudoalteromonas* MB41 performed with fastANI. Download Text S3, TXT file, 0.002 MB. Copyright © 2023 Ohdera et al. 2023 Ohdera et al. https://creativecommons.org/licenses/by/4.0/ This content is distributed under the terms of the Creative Commons Attribution 4.0 International license.

10.1128/msphere.00315-22.10 TEXT S4 Results of the average nucleotide identity analysis for *Vibrio* MA64 performed with fastANI. Download Text S4, TXT file, 0.02 MB. Copyright © 2023 Ohdera et al. 2023 Ohdera et al. https://creativecommons.org/licenses/by/4.0/ This content is distributed under the terms of the Creative Commons Attribution 4.0 International license.
