# Supplementary figures and images for: Comparative Genomic Insights into Bacterial Induction of Larval Settlement and Metamorphosis in the Upside-Down Jellyfish Cassiopea
Source: mSphere. 2023 May 8;8(3):e00315-22. doi: 10.1128/msphere.00315-22 (PMC10286705; doi:10.1128/msphere.00315-22)

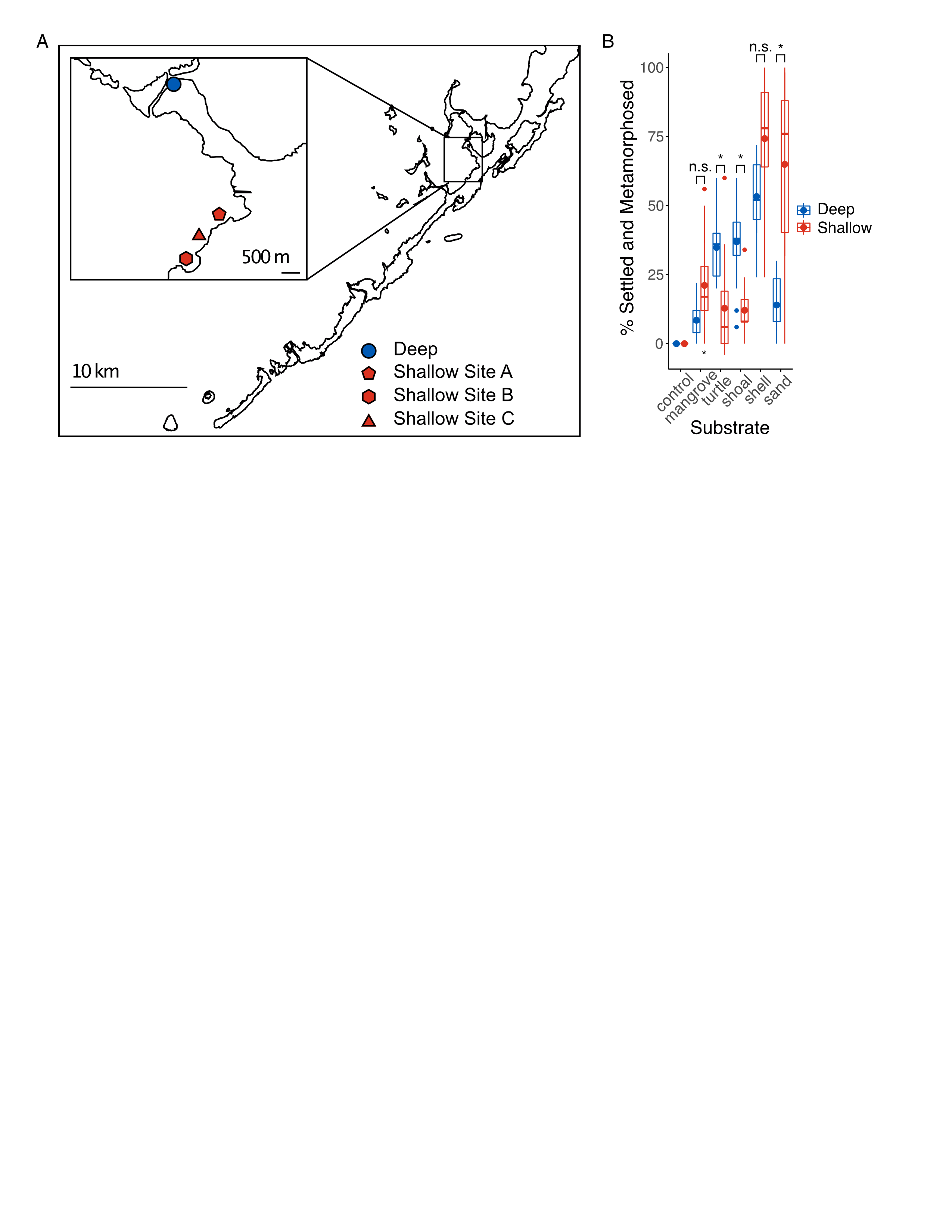

Supplement: FIG S1 [file msphere.00315-22-s0005.tif]

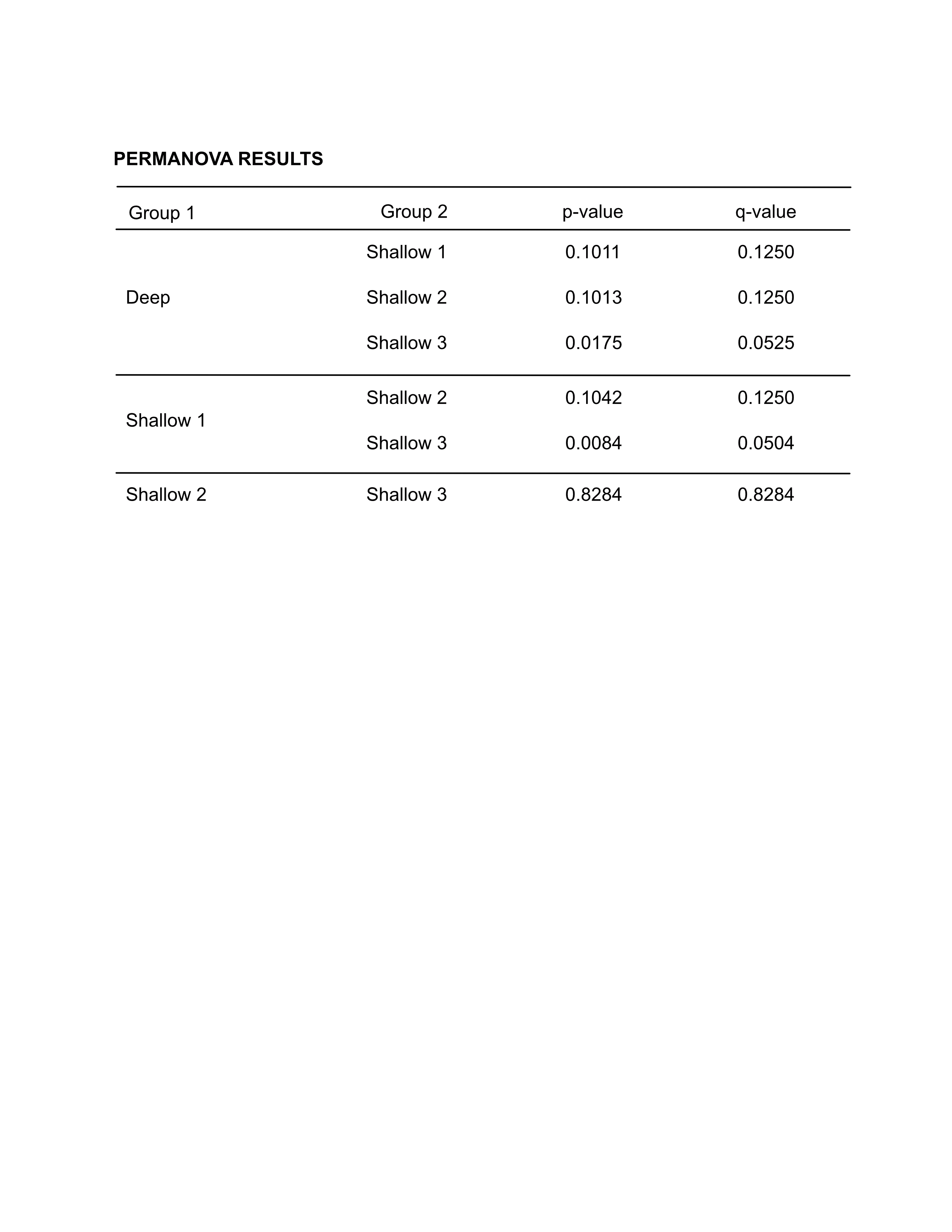

Supplement: TABLE S1 [file msphere.00315-22-s0007.tif]

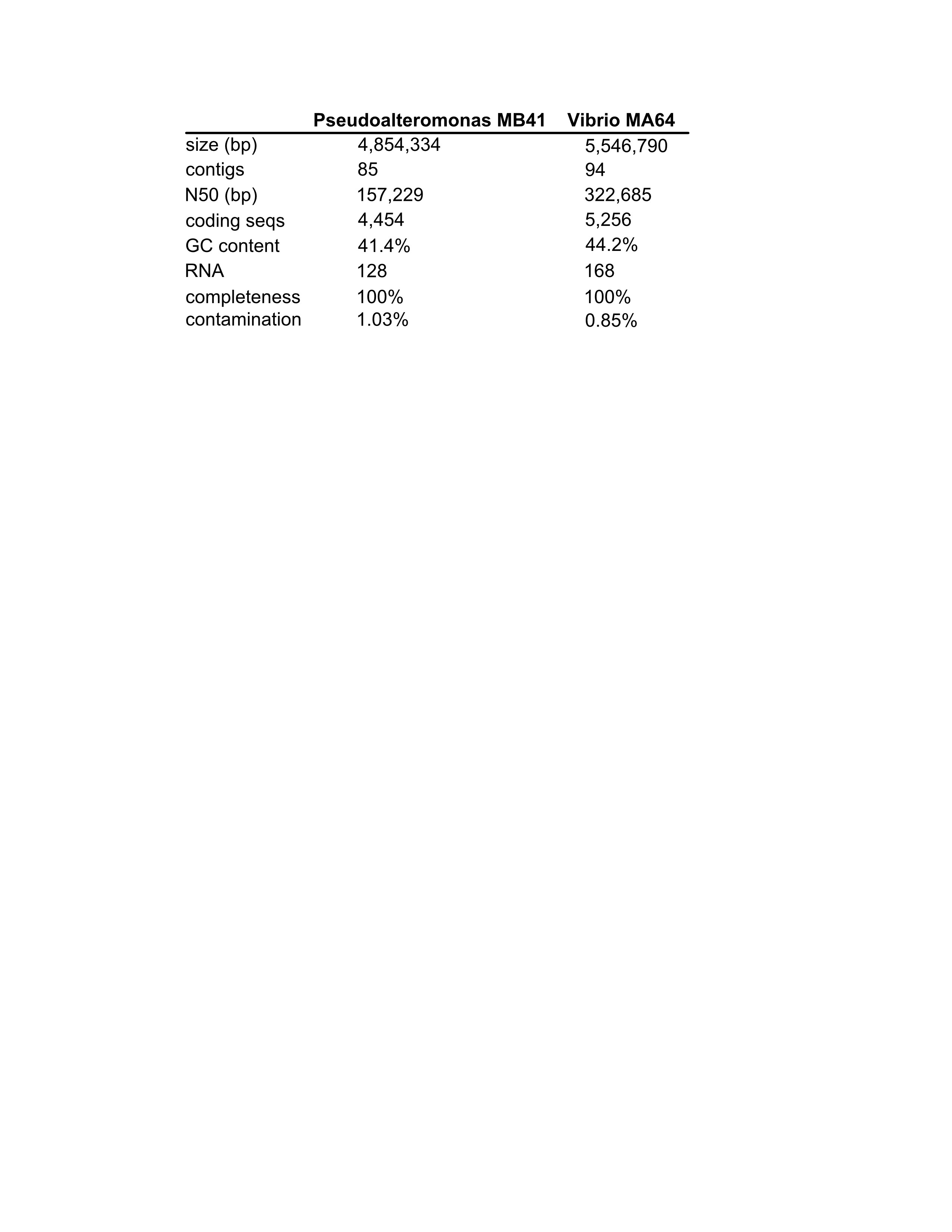

Supplement: TABLE S2 [file msphere.00315-22-s0008.tif]

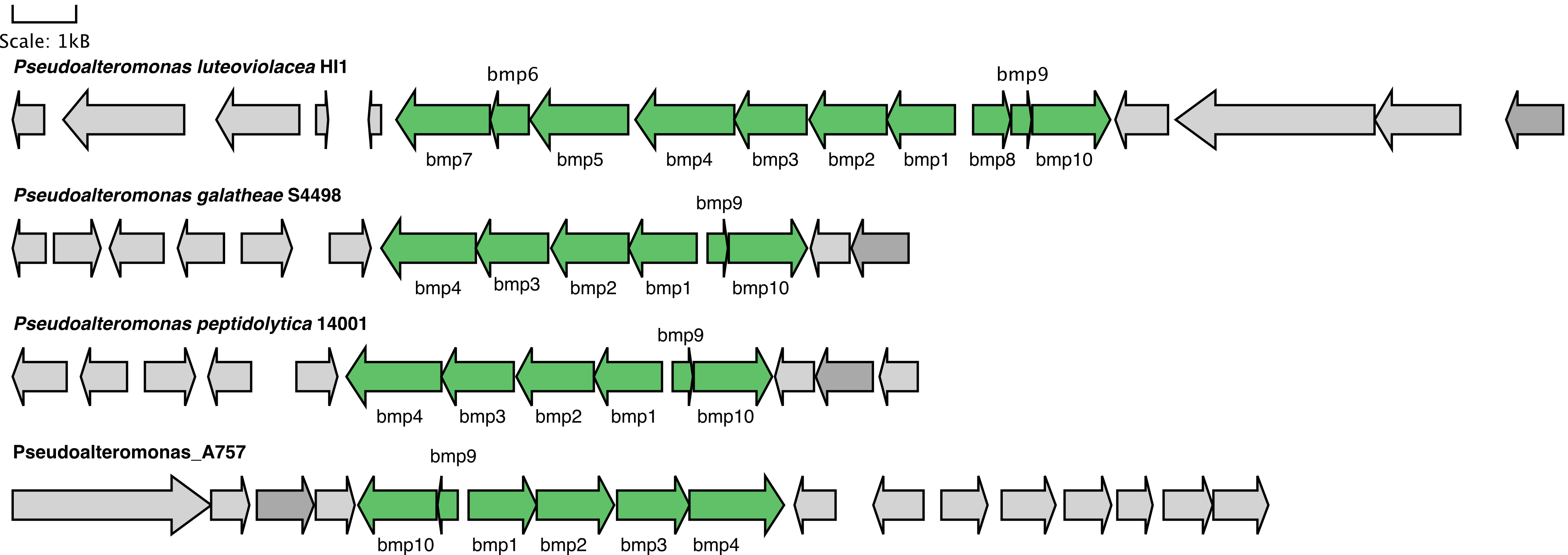

Supplement: FIG S2 [file msphere.00315-22-s0006.tif]

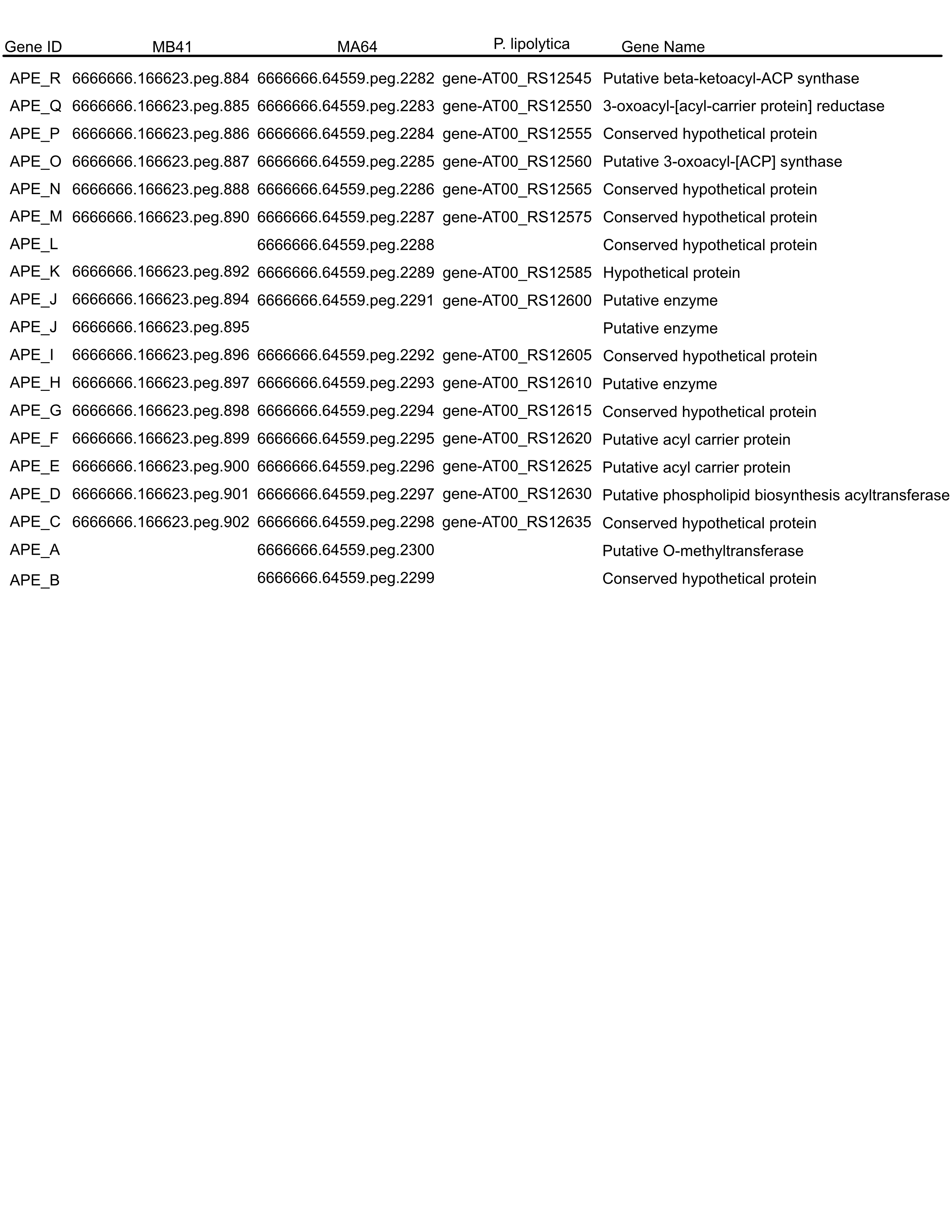

Supplement: TABLE S3 [file msphere.00315-22-s0009.tif]

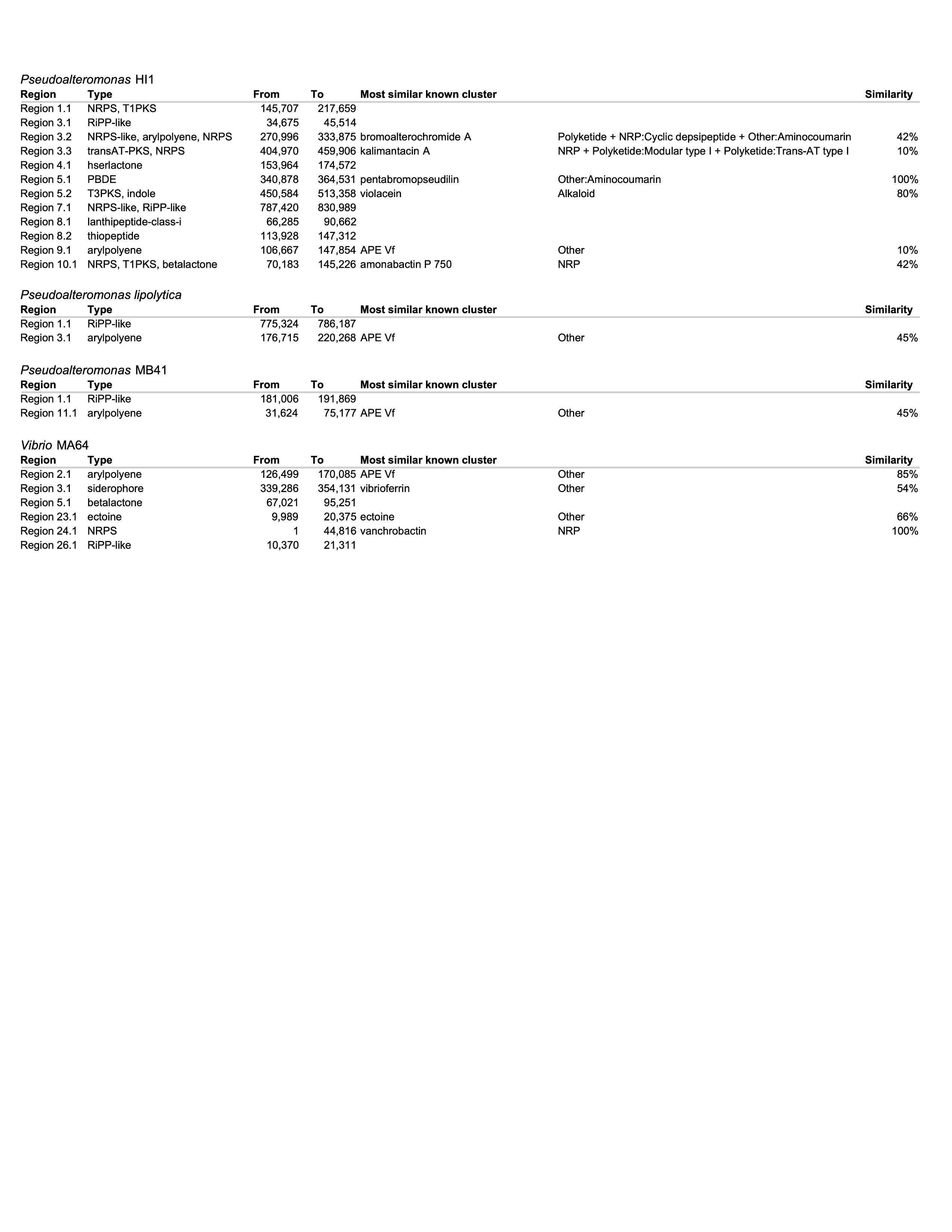

Supplement: TABLE S4 [file msphere.00315-22-s0010.tif]
